# An unusual cutaneous infection caused by *Mycobacterium marinum*

**DOI:** 10.1099/jmmcr.0.005088

**Published:** 2017-04-12

**Authors:** Federica Veronese, Elisa Zavattaro, Pamela Farinelli, Enrico Colombo, Paola Savoia

**Affiliations:** ^1^​ Department of Health Science, University of Eastern Piedmont, Novara, Italy; ^2^​ Department of Translational Medicine, University of Eastern Piedmont, Novara, Italy; ^3^​ SCDU Dermatology, AOU Maggiore della Carità, Novara, Italy

**Keywords:** disease/indication: atypical mycobacteriosis, pathology/symptoms: *Mycobacterium marinum*, treatment: claritrhomycin

## Abstract

**Introduction.** *Mycobacterium marinum* is a non-tubercular mycobacterium residing in fresh or salt water (in tropical or temperate areas); it is a fish and human pathogen, and in immunocompromised patients can cause severe cutaneous and subcutaneous infections.

**Case presentation.** A 46-year-old white man who underwent immunosuppressive therapy was admitted to our department in May 2016 for skin lesions previously diagnosed as ‘unusual erysipelas’. We rejected the hypothesis of erysipelas, due to the clinical features, and our diagnostic hypotheses were oriented towards sporotrichosis, atypical mycobacteriosis, cutaneous tuberculosis and cutaneous sarcoidosis. Histological examination performed after a skin biopsy was compatible with a diagnosis of sporotrichosis. However, PCR performed on fresh tissue demonstrated the presence of *M. marinum*.

**Conclusion.** The case reported is interesting for the unusual clinical localization and modality of infection. The patient became infected by contact with contaminated remains or in the sea, in a geographical area not endemic for *M*. *marinum*. The previous state of immunosuppression favoured infection; however, the presence of *M. marinum* in this area suggests a possible tropicalization of the water of the Mediterranean Sea. To our knowledge, this case is the only one reported in the literature with this modality of infection and in that geographical area.

## Introduction

*Mycobacterium marinum* is a non-tubercular mycobacterium residing in fresh or salt water (in tropical or temperate areas); it is a fish and human pathogen, and in immunocompromised patients can cause severe cutaneous and subcutaneous infections [1]. Herein, we present a case of unusual cutaneous infection caused by *M. marinum*.

## Case report

A 46-year-old white man was admitted to our department in May 2016 for skin lesions previously diagnosed as ‘unusual erysipelas’. The patient was affected by hypogammaglobulinaemia and renal sarcoidosis, and previously underwent immunosuppressive therapy based on tacrolimus and monthly doses of intravenous immunoglobulin.

**Fig. 1.**
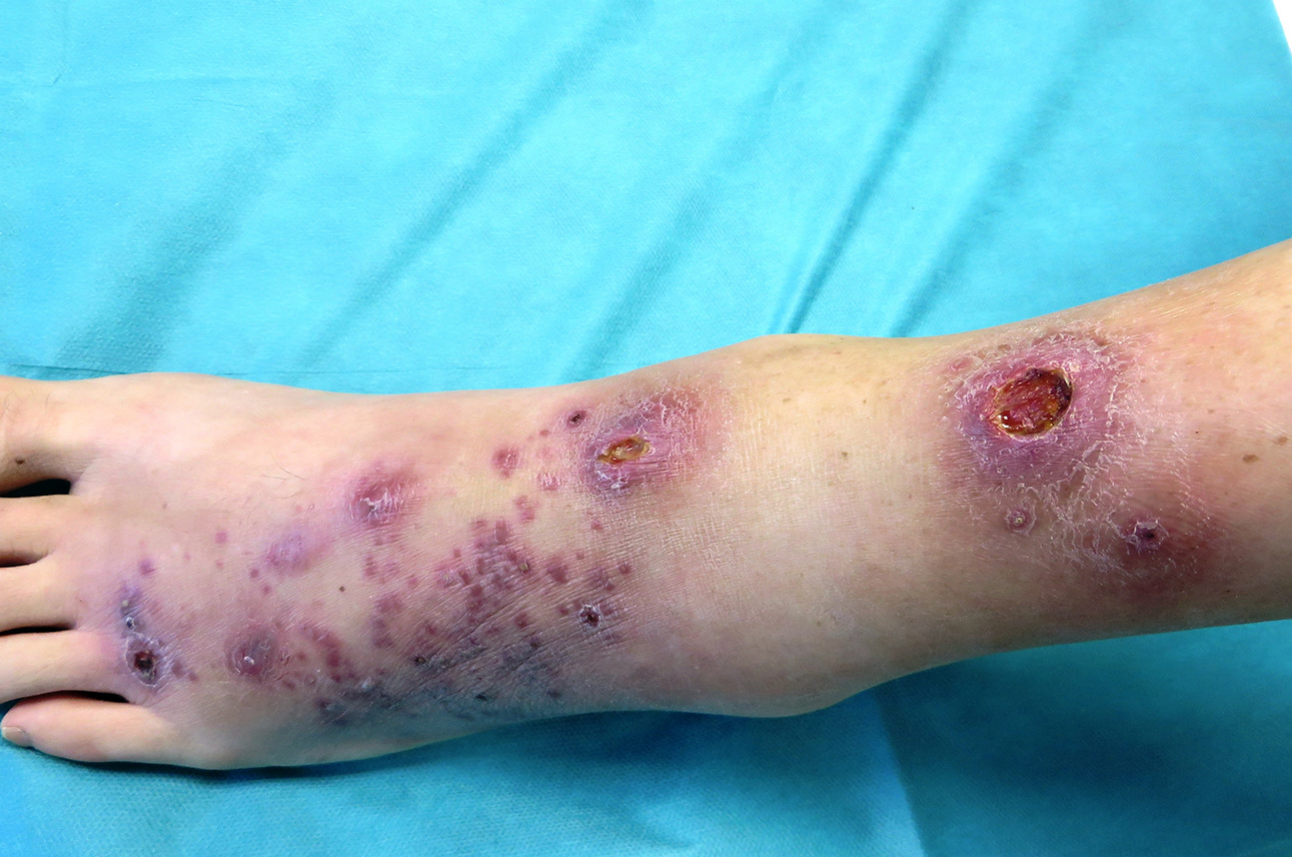
Spread purplish papular-pustular lesions, tending to confluence at the back of the left foot, and two ulcerating nodules with purplish and infiltrated margins on the lower third of the left leg.

Dermatological examination revealed spread purplish papular-pustular lesions, tending to confluence at the back of the left foot, and two ulcerating nodules with purplish and infiltrated margins on the lower third of the left leg (Fig. 1). The patient also presented a satellite nodule and lymphangitic streak on the left thigh. The lesions occurred from September 2015 onwards.

Laboratory examinations gave normal results, with the exception of a slight increase of C-reactive protein and a skin swab positive for *Staphylococcus aureus* sensitive to meticillin and *Streptococcus anginosus.* At the time of the first visit, the patient had already been treated according to antibiogram results with a partial improvement.

We rejected the hypothesis of erysipelas, due to the clinical features, and considered the skin swab positivity as a possible contamination. Our diagnostic hypotheses were oriented towards sporotrichosis, atypical mycobacteriosis, cutaneous tuberculosis and cutaneous sarcoidosis.

Chest X-ray and ultrasound examination did not demonstrate visceral involvement. Histological examination performed after a skin biopsy revealed a pseudoepitheliomatous hyperplasia of the epidermis and a dermic granulomatous inflammatory infiltrate with the formation of epithelioid granulomas. These findings were compatible with a diagnosis of sporotrichosis, but PAS staining was negative. The pathologist also had used Ziehl–Neelsen staining with negative results. However, PCR performed on fresh tissue demonstrated the presence of *M. marinum*. At this point, deepening the medical history, the patient remembered that in the summer of 2015 he had suffered an abrasion under the left foot when walking on the beach in Tuscany (Castiglione della Pescaia). The lesions occurred some months after.

**Fig. 2.**
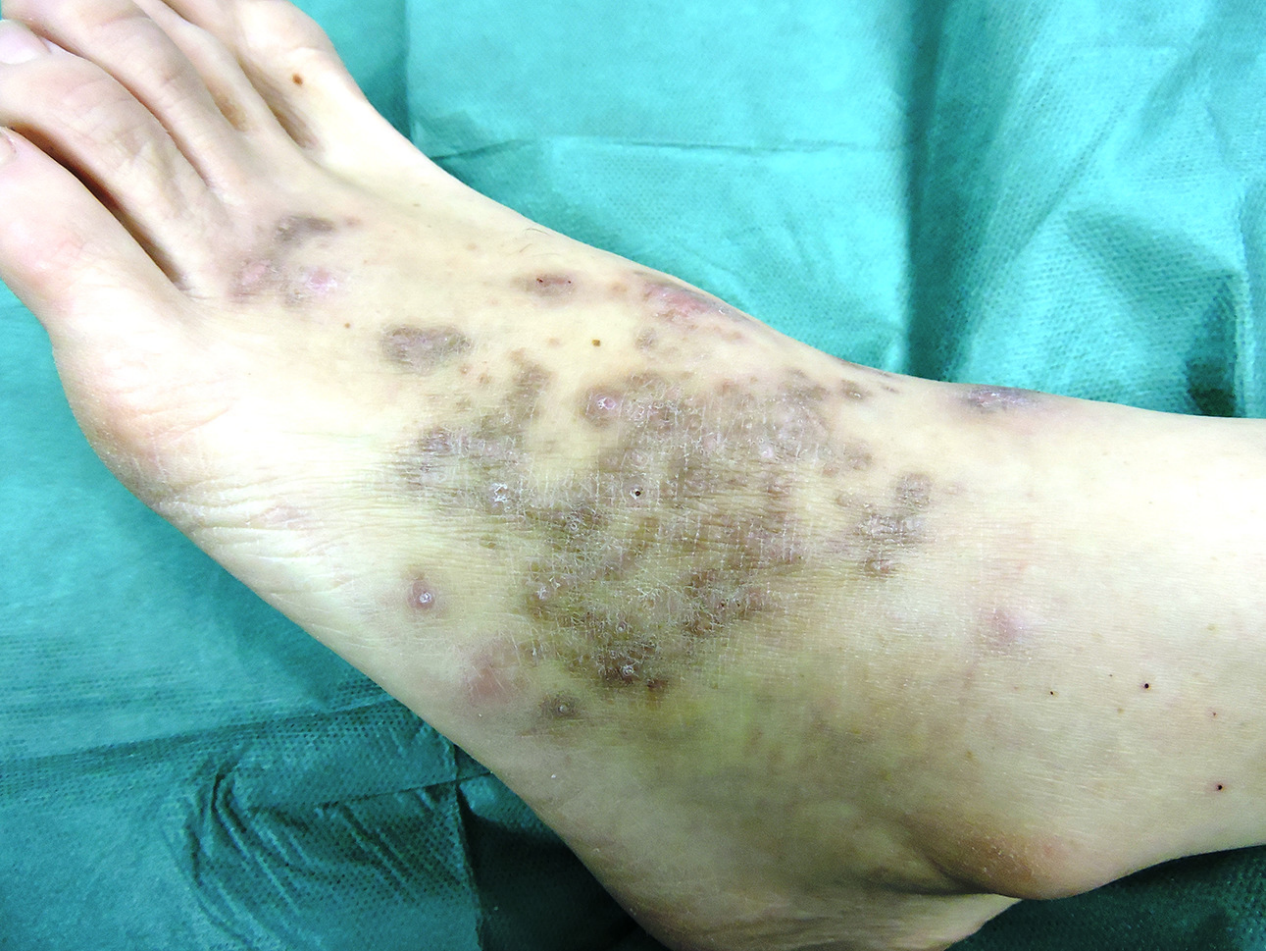
The patient's foot after 6 months of therapy.

The patient was treated with clarithromycin, 500 mg twice daily, and rifampicin, 600 mg once a day orally. After 6 months of therapy, we observed a full clinical, histologically confirmed, remission of the cutaneous lesions. Only a few pigmented, non-palpable lesions remained (Fig. 2).

## Discussion

*M. marinum* is a slow-growing (5–14 days) photochromogens mycobacteria (group I of Runyon’s classification) widely disseminated worldwide [1]. The incubation period for the lesions caused by this bacterium is variable from a week to months (8–60 days) [2]. The median time to diagnosis is 3.5 months [3]. The annual incidence is estimated at 0.27 cases per 100 000 persons in the USA [3], but in the literature there is no data available for the incidence in Italy.

The common presentation in immunocompetent patients is generally related to jobs or hobbies with exposure to fresh or salt water; indeed, the main form of inoculation is skin trauma followed by exposure to the marine environment or aquatic animals (fish, shell, aquariums) [1]. Thus, *M. marinum* infections are known as ‘fish-tank granuloma’, ‘aquarium granuloma’ or ‘swimming pool granuloma’, although the frequency of such manifestations is reduced due to the chlorination of the water [1, 2]. The more frequently affected anatomical sites are the hands, forearms and elbows [2]. Initially, a solitary, violaceous papule or nodule at the site of inoculation can progress to a verrucous plaque and/or ulcer. Proximal extension may occur through lymphatic spread and 20 % of patients present with a sporotrichoid distribution [1], particularly in immunosuppressed patients who may have scattered and deep manifestations with septicaemia [4]. The main immunosuppressive conditions predisposing to atypical mycobacteriosis are human immunodeficiency virus infection, solid organ transplantation (incidence of 0.2–2.8 % in heart, 0.5–8 % in lung and 0.16–0.38 % in kidney transplant patients [5]) and therapy with TNF inhibitors (infliximab, adalimumab) [3, 6].

For the correct diagnosis, the following are important: a careful medical history, physical and histological examination, and microbiological culture on fresh tissue from skin biopsy. PCR on tissue or culture is very helpful to confirm diagnosis [7].

The therapy varies depending on the severity of the lesions; if the lesions are limited, they can resolve spontaneously or with antibiotic monotherapy (clarithromycin, azithromycin, moxifloxacin, tetracyclines and trimethoprim/sulfamethoxazole) [1]. Multidrug therapy is recommended for more extensive infections with the involvement of deeper structures or for severe infections with a sporotrichoid distribution pattern (clarithromycin associated with rifampicin or ethambutol) [1, 6]. The treatment is prolonged, taking between 6 weeks and 6 months (at least 2 months after the clinical remission [6] and in deep infections taking 18 months [4, 8]). Surgical treatment is usually reserved for cases of subcutaneous tissue (tendons or bone) involvement [1].

The case reported is interesting for the unusual clinical localization and modality of infection. The patient became infected by contact with contaminated remains or in the sea, in a geographical area not endemic for this bacterium. The previous state of immunosuppression favoured infection; however, the presence of *M. marinum* in this area suggests a possible tropicalization of the water of the Mediterranean Sea. To our knowledge, this case is the only one reported in the literature with this modality of infection and in that geographical area.
